# An explosive innovation: Phylogenetic relationships of *Solanum* section *Gonatotrichum* (Solanaceae)

**DOI:** 10.3897/phytokeys.8.2199

**Published:** 2012-01-05

**Authors:** Stephen Stern, Lynn Bohs

**Affiliations:** 1 Department of Biology, University of Utah, 257 South 1400 East, Salt Lake City, Utah, 84112-0840, USA; 2 Department of Biology, Colorado Mesa University, 1100 North Ave., Grand Junction, CO, 81501, USA

**Keywords:** explosive fruit dehiscence, Neotropics, *Solanum*, phylogeny

## Abstract

*Solanum* is one of the largest plant genera and exhibits a wide range of morphological diversity. *Solanum* section *Gonatotrichum*, the focus of this study, is unique within the genus because of its fruits that swell with turgor pressure and explosively dehisce to disperse the seeds. We infer phylogenetic relationships within section *Gonatotrichum* using DNA sequence data from two nuclear regions (ITS and the granule-bound starch synthase gene [GBSSI or *waxy*]) and the chloroplast region *trnT-F*. The resulting phylogenetic trees support the monophyly of the section with the inclusion of *Solanum lignescens*, a species not previously thought to belong to the group due to the presence of stellate hairs. This inclusion of this species in section *Gonatotrichum* suggests that the simple, often geniculate hairs of species in the group may represent reduced stellate hairs. The presence of heterantherous flowers appears to be derived in the section, but this character is largely lost in *Solanum parcistrigosum*.

## Introduction

*Solanum* (Solanaceae), with approximately 1500 species, is one of the 10 largest flowering plant genera (Frodin 2004; Bohs 2005). Recent species-level taxonomy (Knapp et al. 2004; http://www.solanaceaesource.org) and numerous molecular phylogenetic studies (e.g., Bohs 2005; Weese and Bohs 2007) have helped to define infrageneric groups within the genus, some of which correspond to formally named subgenera or sections. Weese and Bohs (2007) recognized 12 to 15 major clades in *Solanum*, one of which they called the Brevantherum clade. This large clade includes species with short, broad anthers that lack spines but commonly have stellate hairs or lepidote scales. It encompasses members of the formally named sections *Brevantherum* Seithe, *Extensum* D’Arcy, *Lepidotum* Seithe, *Stellatigeminatum* A. Child, and *Gonatotrichum* Bitter. *Solanum* section *Cernuum* Carvalho & G. J. Sheph. belongs to the clade on the basis of morphological data, but no sequence data have been available to place it in a molecular phylogeny.

*Solanum* section *Gonatotrichum* is morphologically unusual within the Brevantherum clade because the species traditionally placed within it have simple rather than stellate hairs and unique fruits with explosive dehiscence (see below; Bitter 1912; Nee 1989, 1999; Weese and Bohs 2007). Prior to molecular studies, its affinities were thought to be with the Morelloid clade of the non-spiny Solanums, not with the Brevantherum clade (D’Arcy 1972, 1991; Nee 1999; Child and Lester 2001). The taxonomy of section *Gonatotrichum* has been poorly understood and its sectional limits have been unclear. Until recently, this section was thought to contain two species (Nee 1989). A forthcoming revision (Stern et al. in review) and this contribution recognize eight species within it: *Solanum adscendens* Sendtn., *Solanum deflexum* Greenm., *Solanum evolvuloides* Giacomin & Stehmann (recently described, see Giacomin and Stehmann 2011), *Solanum hoffmanseggii* Sendtn., *Solanum lignescens* Fernald, *Solanum manabiense* S. Stern, *Solanum parcistrigosum* Bitter, and *Solanum turneroides* Chodat. The purpose of this study is to clarify the circumscription of section *Gonatotrichum* and to investigate the phylogenetic relationships of the species of the section as well as its placement within the larger Brevantherum clade.

Species in section *Gonatotrichum* are native to North, Central, and South America. They are herbs or small, woody shrubs with short inflorescences and simple hairs (except *Solanum lignescens*, which has stellate pubescence; see below). Flowers in the section have corollas ranging from 1–2.5 cm in diameter. The largest flowers are those of *Solanum turneroides* and *Solanum evolvuloides*, which also exhibit marked heteranthery, in which one filament is nearly double the length of the other four. The fruits of species in section *Gonatotrichum* are unique within the genus. They have a thin pericarp with a watery mesocarp held under pressure until they explosively dehisce. The fruits are white, yellow, or green, nearly transparent, and turgid before explosive dehiscence and deflated and shriveled after dehiscence.

Some species within section *Gonatotrichum* are relatively widespread (*Solanum deflexum*, *Solanum parcistrigosum*, and *Solanum turneroides*), whereas others are narrowly distributed and relatively inconspicuous, making them among the least collected species of *Solanum*. As Bitter (1912) noted in the original description of the section, the group has a large geographic disjunction, with species found in the southwestern USA, Mexico, and Central America as far south as Costa Rica and then again in southern South America (Bolivia, Paraguay, northwestern Argentina, and southern Brazil). The recent description of *Solanum manabiense* from coastal Ecuador (Stern and Bohs 2009) and identification of previously undetermined specimens, particularly those of *Solanum hoffmanseggii* from Pará and Tocantins states in Brazil, has lessened the area of this disjunction; however, the section is absent from large areas of South America. This is likely due to the preference of the species for lower-altitude, dry habitats that are widely spaced on the continent.

Species placed in section *Gonatotrichum* have been subjected to two previous phylogenetic studies. Bohs (2005) and Weese and Bohs (2007) obtained sequence data for three species of the section (*Solanum deflexum*, *Solanum turneroides*, and “*Solanum adscendens*”, later reidentified as *Solanum parcistrigosum*). In both studies they formed a strongly supported monophyletic group sister to the remaining sampled members of the Brevantherum clade. Concurrently, morphological and field studies of the group identified two new species, *Solanum manabiense* (Stern and Bohs 2009) and *Solanum evolvuloides* (Giacomin and Stehmann 2011), and clarified species limits and nomenclatural problems in the section. The presence of explosively dehiscent fruits in the Mesoamerican *Solanum lignescens* suggested that it also belongs to the group despite its stellate rather than simple pubescence (Stern et al. in review).

In this paper we use molecular phylogenetic methods to 1) examine the phylogenetic relationships of section *Gonatotrichum* with other members of the genus, 2) test the monophyly of section *Gonatotrichum*, 3) test the monophyly of species within the section and examine selected species-level relationships, and 4) examine geographical distributions and morphological patterns within the section.

## Materials and methods

### Taxon sampling

Seven of the eight recognized species of section *Gonatotrichum* were sampled. We were unable to obtain high quality genomic DNA for *Solanum hoffmanseggii*, an undercollected species from Amazonian Brazil. Multiple accessions were sampled for four species, with four accessions sampled for *Solanum parcistrigosum*, and two each for *Solanum evolvuloides*, *Solanum manabiense*, and *Solanum deflexum*. In addition to the species of section *Gonatotrichum*, we included members of *Solanum* as outgroups guided by results from previous studies showing other members of the Brevantherum clade to be sister to the section (Bohs 2005; Weese and Bohs 2007). Members of section *Geminata* (G. Don) Walp. were included as a more distant outgroup and the tree was rooted with *Solanum betaceum*, a member of section *Pachyphylla* (Dunal) Dunal. All taxa, along with voucher information and GenBank accession numbers, are listed in Appendix 1.

### DNA Extraction, amplification and sequencing

Total genomic DNA was extracted from fresh, silica gel-dried, or herbarium material using the DNeasy plant mini extraction kit (Qiagen, Inc., Valencia, California). PCR amplification for each gene region followed standard procedures described in Taberlet et al. (1991), Bohs and Olmstead (2001), and Bohs (2004) for the *trnT-L* and *trnL-F* intergeneric spacer regions; Levin et al. (2005) for *waxy*; and Levin et al. (2006) for ITS. The ITS region was amplified as a single fragment using primers ITSleu1 (Bohs and Olmstead 2001) and ITS4 (White et al. 1990) using PCR conditions described in Bohs and Olmstead (2001). When possible, *trnT-F* and *waxy* were amplified as single fragments using primers a and f for *trnT-F* (Taberlet et al. 1991) and primers waxyF and waxy2R for *waxy* (Levin et al. 2005). PCR conditions for *trnT-F* followed Bohs and Olmstead (2001); conditions for *waxy* followed Levin et al. (2005). When necessary, overlapping fragments were amplified and assembled, using primers a with d and c with f to amplify *trnT-F*, and primers waxyF with 1171R, and 1058F with 2R to amplify *waxy*.

PCR products were cleaned using the Promega Wizard SV PCR Clean-Up System (Promega Corporation, Madison, Wisconsin). The University of Utah DNA Sequencing Core Facility performed sequencing on an ABI automated sequencer. Sequences were edited in Sequencher (Gene Codes Corp., Ann Arbor, Michigan) and all new sequences were submitted to GenBank; accession numbers are listed in Appendix 1.

### Sequence alignment and analysis

Sequence alignment for all of the gene regions was straightforward and performed visually using Se-Al (Rambaut 1996). The aligned dataset is available as Appendix 2 (see Appendix 2: Aligned Dataset).

### Parsimony analyses

Parsimony analyses were performed on each dataset separately and on the combined dataset using PAUP*4.0b10 (Swofford 2002). All characters were weighted equally in analyses that implemented TBR branch swapping with 1,000 heuristic random addition replicates, each limited to 1,000,000 swaps per replicate. Gaps were treated as missing data. Bootstrapping (BS; Felsenstein 1985) was used to evaluate branch support with 1,000 random addition replicates and TBR branch swapping limited to 1,000,000 swaps per replicate.

### Bayesian analyses

Prior to Bayesian analyses, a general model of nucleotide evolution was selected for the separate and the combined datasets using the AIC criterion identified in Modeltest 3.7 (Posada and Crandall 1998). MrBayes 3.1 (Hulsenbeck and Ronquist 2001) was used to analyze each of the separate and combined datasets. For each analysis, five million generations were run usingeightMarkov chains, each initiated from a random tree and sampled every 1,000 generations. Each of the analyses reached a standard deviation below 0.01 between the chains and all parameters from each analysis were visualized graphically to determine the trees discarded as burn-in prior to achieving stationarity.

## Results

### Phylogenetic analyses

The parsimony strict consensus and Bayesian majority rule consensus trees of all datasets differed only in the degree of resolution, with Bayesian tree topologies more resolved than parsimony trees (Table 1). Clades with low posterior probabilities, typically those below 0.90 PP but occasionally those with up to 1.0 PP in Bayesian analyses were often collapsed in parsimony strict consensus trees. Descriptive statistics for individual and combined genes are provided (Table 1). More nodes were strongly supported by combining the data than were obtained in any of the separate analyses.

**Table 1.** Descriptive statistics for each data set analyzed. Strongly supported nodes for parsimony indicate those with ≥ 90% BS; Bayesian strongly supported nodes are those with ≥ 0.95 PP.

**Data Partition**	**Aligned Sequence Length**	**# Parsimony Informative Characters**	**# MP Trees**	**Tree Length**	**CI**	**RI**	**# Strongly Supported Nodes Parsimony**	**Model Selected**	**# Strongly Supported Nodes Bayesian**
ITS	709	127	14	435	0.632	0.683	4	GTR+I+G	15
waxy	2090	173	40	403	0.893	0.942	10	TIM+G	21
trnT-F	1953	71	>135,000	178	0.944	0.960	8	TVM+G	12
combined	4752	371	14	1031	0.779	0.847	13	GTR+I+G	25

### Topological conflicts

**Figure 1.**
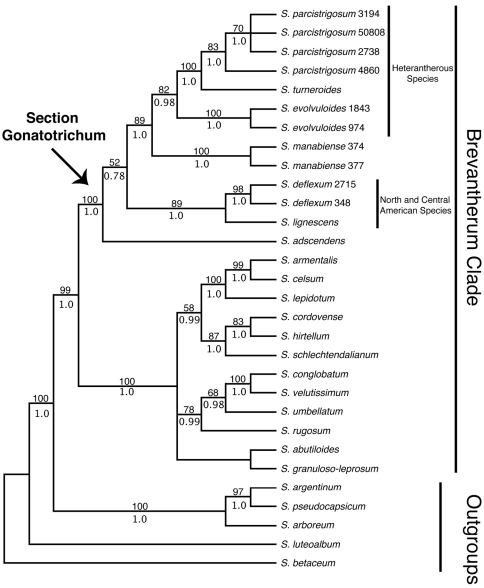
Strict consensus of the 14 most parsimonious trees from the concatenated MP analysis of ITS, *trnT-F*, and *waxy* data. Bootstrap values > 50% and posterior probabilities are shown above and below the branches, respectively. Numbers after species names indicate collector numbers listed in Appendix 1 for multiple accessions of a single species.

**Figure 2.**
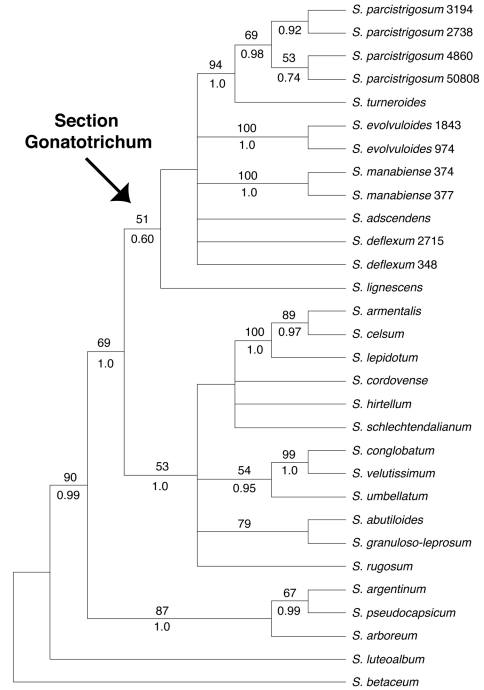
Strict consensus of the 14 most parsimonious trees from the MP analysis of the ITS dataset. Bootstrap values > 50% and posterior probabilities are shown above and below the branches, respectively.

**Figure 3.**
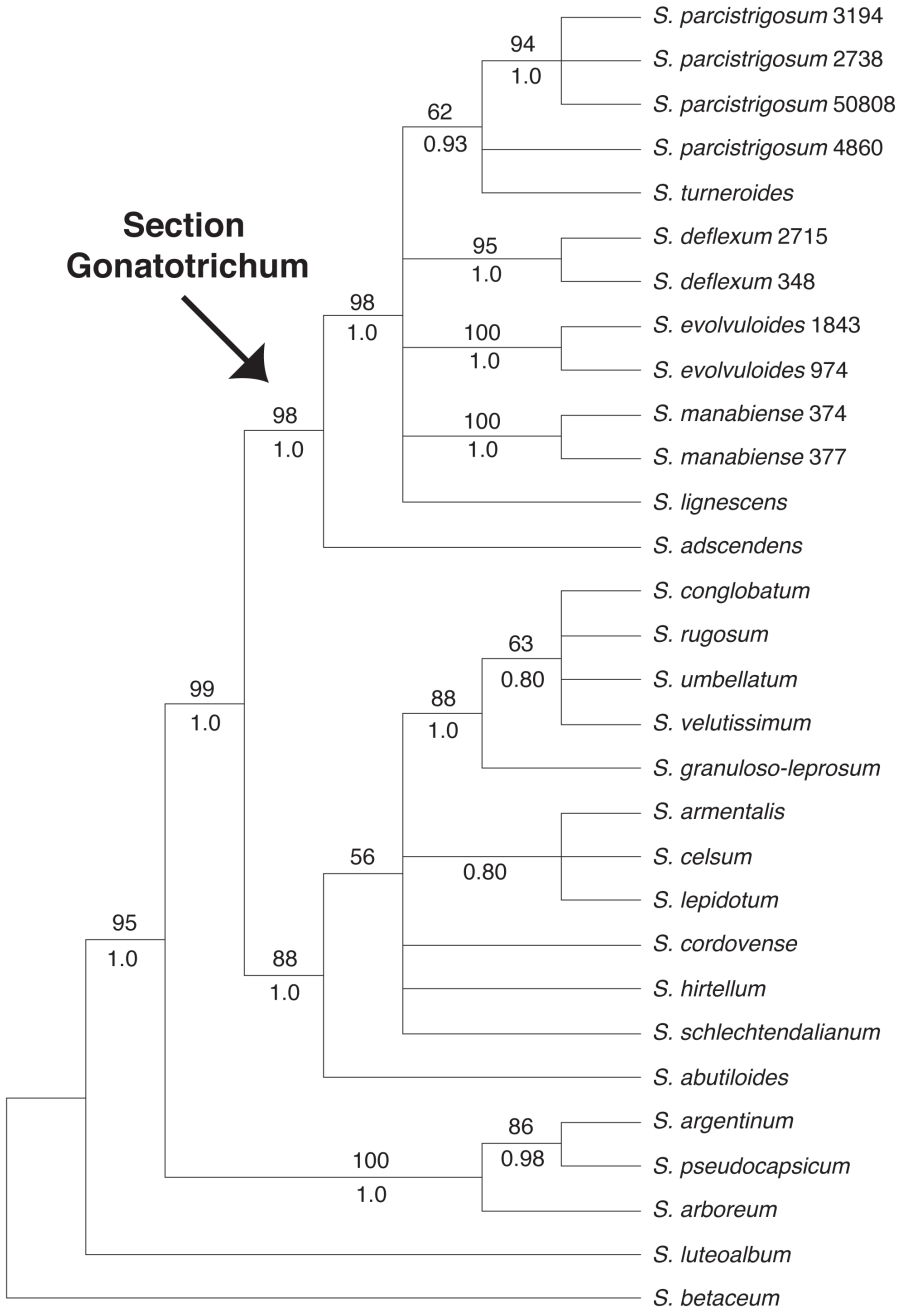
Strict consensus of the more than 135,000 most parsimonious trees from the MP analysis of the *trnT-F* dataset. Bootstrap values > 50% and posterior probabilities are shown above and below the branches, respectively.

**Figure 4.**
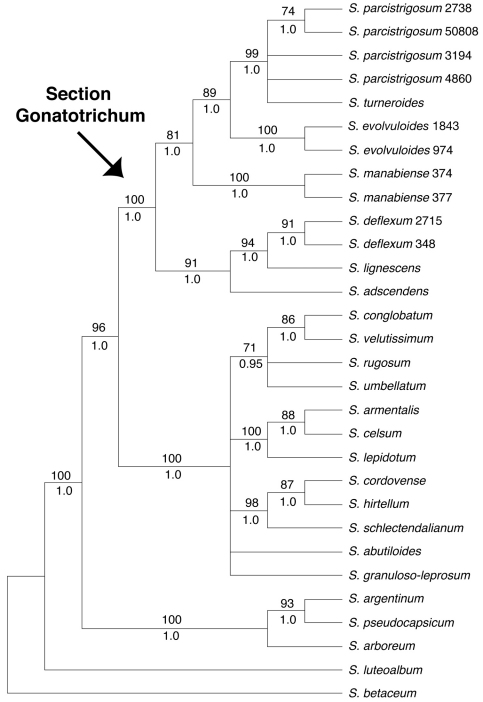
Strict consensus of the 40 most parsimonious trees from the MP analysis of the *waxy* (GBSSI) dataset. Bootstrap values > 50% and posterior probabilities are shown above and below the branches, respectively.

Our discussion will largely be based on the parsimony strict consensus tree of the combined data set, which is a conservative hypothesis of phylogenetic relationships, but areas of the tree that receive strong support in the Bayesian analysis that are less strongly supported in the parsimony analysis will be noted (Fig. 1). The parsimony strict consensus trees for the individual markers are also presented (Fig. 2–4). In parsimony analyses, each DNA sequence region consistently identified the same major, well-supported groups corresponding to the Brevantherum clade and section *Gonatotrichum* comprising identical species, but relationships within these major clades were often not strongly supported (BS values < 90 %), or were unresolved, and thus cannot be considered conflicting under Wiens’ (1998) criteria. Within section *Gonatotrichum*, one incongruence of note among the various datasets is the placement of *Solanum adscendens*, *Solanum lignescens*, and *Solanum deflexum*. In the plastid (Fig. 3) and combined trees (Fig. 1), *Solanum adscendens* is sister to the remainder of the species in the section (98% BS, 1.0 PP in the *trnT-F* tree, 52% BS, 0.78 PP in the combined tree). In the ITS tree, *Solanum lignescens* is sister to the remaining species in section *Gonatotrichum*, but this relationship is unsupported (Fig. 2). In the *waxy* tree, *Solanum adscendens*, *Solanum lignescens*, and *Solanum deflexum* form a well-supported clade (91% BS, 1.0 PP) sister to the remaining species of section *Gonatotrichum* (Fig. 4).

### Phylogenetic relationships

*Solanum* section *Gonatotrichum* emerges as monophyletic in all analyses and strongly supported in all except the ITS-only tree. The section is strongly supported (99% BS, 1.0 PP in the combined tree) as sister to the remainder of the species sampled from the Brevantherum clade, which form a monophyletic group in all analyses (100% BS, 1.0 PP in the combined tree; for sampling of Brevantherum clade see Fig. 1). Species with duplicate accessions sequenced (*Solanum parcistrigosum*, *Solanum evolvuloides*, *Solanum manabiense*, and *Solanum deflexum*) were all monophyletic in the combined tree. Within section *Gonatotrichum*, *Solanum turneroides* is strongly supported as sister to *Solanum parcistrigosum* in the combined tree (100% BS, 1.0 PP), and *Solanum evolvuloides* is sister to this clade (82% BS, 0.98 PP). *Solanum manabiense* from coastal Ecuador is resolved as sister to this clade (89% BS, 1.0 PP). *Solanum deflexum* and *Solanum lignescens* form a clade (89% BS, 1.0 PP). The final species, *Solanum adscendens*, is sister to the remainder of the species of section *Gonatotrichum* in the combined tree, although this relationship is poorly supported (52% BS, 0.78 PP) and, as noted above, is not recovered in the ITS and *waxy* trees.

## Discussion

Our data, like those of previous studies, show that *Solanum* section *Gonatotrichum* belongs to the Brevantherum clade (sensu Bohs 2005; Weese and Bohs 2007). This clade consists of approximately 60 species of herbs, shrubs and trees found in tropical and subtropical regions of the New World (Bohs 2005). Centers of diversity occur in the Andes and in eastern Brazil, where many species are common in secondary vegetation and disturbed habitats. All members of the clade lack prickles and have relatively short, broad anthers. However, members of section *Gonatotrichum* are strikingly different from the rest of the species of the Brevantherum clade. Traditionally, the section included small annuals or perennials with reduced inflorescences and simple hairs, whereas the remainder of the Brevatherum clade includes shrubs to sizable woody trees with stellate or lepidote pubescence and often large, branched inflorescences. The explosively dehiscent fruits of section *Gonatotrichum* differ from those of the rest of the Brevantherum clade, which are variously colored, fleshy in texture, and not dehiscent at maturity.

These results indicate that section *Gonatotrichum* forms a monophyletic group including the Mesoamerican *Solanum lignescens*. This species had not been considered to be a member of the section *Gonatotrichum* in previous taxonomic treatments (Bitter 1912, 1913; Nee 1989). Nee (1999) placed *Solanum lignescens* in section *Brevantherum* due to its shrubby habit and stellate hairs, although he indicated that it had no obvious close relatives within the section and suggested that perhaps it might belong in section *Gonatotrichum*. Our molecular data place *Solanum lignescens* within section *Gonatotrichum* and, in the combined and *waxy* trees, as sister to *Solanum deflexum*, a species with exclusively simple hairs found from the southwestern USA to Mexico and Central America. The fruits of *Solanum lignescens* are explosive berries like those of the rest of the section, indicating that fruit morphology may be a synapomorphy for section *Gonatotrichum*, but that habit and hair morphology may be somewhat variable within it.

Hairs have been used extensively for identification of species and sections in *Solanum* and have their own standardized terminology in the genus (see Roe 1971 for a overview). While the presence of specific hair types can be diagnostic, many groups or even species can have multiple hair types. Section *Brevantherum* sensu Roe (1972), placed in the Brevantherum clade by Bohs (2005) and Weese and Bohs (2007), exemplifies this complexity, with some species having up to six different hair types (see Table 1, Roe 1972). Section *Gonatotrichum* was previously distinguished within the genus due to the presence of simple, geniculate hairs with a short basal cell and a 90° bend between this and the second cell in at least some of its members (Bitter 1912, 1913; Nee 1989). Geniculate hairs are present in some species in the section (*Solanum hoffmanseggii* and *Solanum parcistrigosum*) but absent in other species (*Solanum adscendens*, *Solanum deflexum*, *Solanum evolvuloides*, and *Solanum manabiense*) with one species (*Solanum turneroides*) having intermediate hairs that are flattened along the stem but lack a 90° bend between the basal and second cells. Our results show that a species with stellate hairs, *Solanum lignescens*, also belongs in section *Gonatotrichum*, which previously was thought to contain only simple-haired species. The placement of *Solanum lignescens* as sister to the simple-haired *Solanum deflexum* in some analyses suggests that simple hairs may have evolved from branched hairs in the latter species and perhaps in the four species sister to the *Solanum lignescens*/*Solanum deflexum* clade in the combined and *waxy* trees. Further morphological, developmental, and phylogenetic study of various species of the Brevantherum clade may clarify the patterns of hair evolution throughout the group and distinguish between simple hairs that are pleisiomorphic versus those derived via ray reduction from branched-haired ancestors.

We sampled multiple accessions per taxon in four of the seven species of section *Gonatotrichum* included in the phylogeny (*Solanum evolvuloides*, *Solanum deflexum*, *Solanum manabiense*, and *Solanum parcistrigosum*). In the combined and *waxy* trees, all four species were monophyletic. The four accessions of *Solanum parcistrigosum* were not monophyletic in the *trnT-F* tree, and the two accessions of *Solanum deflexum* did not form a clade in the ITS tree. However, neither of these cases of species non-monophyly were strongly supported. It appears that species limits, at least within these four taxa, are fairly distinct.

Within section *Gonatotrichum*, the phylogeny from the combined data exhibits clear geographic patterns. The species ranging from North to Central America (*Solanum lignescens* and *Solanum deflexum*) form a strongly supported clade, as do the southern South American species (*Solanum evolvuloides*, *Solanum parcistrigosum*, and *Solanum turneroides*) with the Ecuadorian *Solanum manabiense* sister to the southern South American species. The position of the Brazilian *Solanum adscendens* as sister to the rest of the clade in the combined and *trnT-F* trees does not fit this biogeographic pattern and is confounded by the incongruence between the *trnT-F* and *waxy* datasets indicated above, although neither of these datasets place *Solanum adscendens* with the remainder of the southern South American species. Future molecular studies should attempt to include *Solanum hoffmanseggii*, a poorly known species from Amazonian Brazil and the only member of section *Gonatotrichum* for which sequence data are not available. Based on morphology, particularly the geniculate hairs that lay flat along the stem, the similar sized flowers and fruits, and the overall distribution, we speculate that *Solanum hoffmanseggii* will likely be closely related to the southern South American species *Solanum parcistrigosum*. Sequence data is needed to confirm this relationship and determine its phylogenetic affinities to the rest of the South American taxa of the clade.

Although buzz pollination is virtually universal in *Solanum*, heteranthery is an unusual trait in the genus and has been shown to have evolved multiple times independently within it (Bohs et al. 2007). It has been particularly well studied in the temperate spiny weed, *Solanum rostratum* Dunal, a member of *Solanum* section *Androceras* (Nutt.) Marzell in the subgenus *Leptostemonum* (Dunal) Bitter (Bowers 1975; Vallejo-Marín et al. 2009). This species has four yellow stamens that serve a “feeding” function, providing pollen that is used as food for the bee pollinator. Pollination is achieved by an elongate, brown lower stamen that is specialized for placing pollen on areas of the insect where it cannot be easily removed and used for food (Vallejo-Marín et al. 2009). In section *Gonatotrichum*, *Solanum evolvuloides* and *Solanum turneroides* are both strongly heterantherous, with the lowermost filament extending to 2-5 mm, thereby reaching double the length of the other stamens. These two species also have flowers that open during the morning hours and close by midday (Nee 1989; S. Stern and L. Giacomin pers. obs.) with the flowers of *Solanum turneroides* also strongly fragrant, an unusual trait in the genus. The pollinators of these species are unknown and it is also unknown whether the upper stamens are modified for “feeding” and the lower stamen modified for “pollinating” as described for *Solanum rostratum* above. The third member of this clade, *Solanum parcistrigosum*, has flowers that are only weakly heterantherous with the filament of the lowermost stamen only ca. 1 mm longer than the other stamens.

Future work on section *Gonatotrichum* should include sequencing of more gene regions and species accessions, especially targeting *Solanum hoffmanseggii* and *Solanum adscendens*, to clarify biogeographic patterns in the group. Nothing is known about chromosome numbers or potential hybridization among taxa, and the function of heteranthery in pollination of several species within the group is unclear. Detailed studies of the development and morphology of hairs in *Solanum* and in section *Gonatotrichum* in particular may reveal that simple hairs may have arisen by two different evolutionary pathways, either from plesiomorphically simple hairs or by reduction from branched hairs. Finally, more in-depth studies of the entire Brevantherum clade are needed to clarify its species limits, phylogenetic relationships, and morphological and biogeographical patterns.

## Appendix 1

**Table:** Summary of species, collection location, vouchers, herbarium acronym, and GenBank accession numbers for taxa used in this study provided in the order ITS, *trnT-F*, and *waxy*. BIRM samples have the seed accession number for the Solanaceae collection at the University of Birmingham, UK (now transferred to the Botanic Garden of the University of Nijmegen, Nijmegen, The Netherlands, http://www.bgard.science.ru.nl/solanaceae/).

**Species**	**Collection Location**	**Voucher and Herbarium Acronym**	**GenebankAccession Number for ITS**	**Genebank Accession Number for *trnT-*F**	**GenBank Accession Number for *wax*y**
***Solanum abutiloides*** (Griseb.) Bitter & Lillo	BIRM S.0655	*Olmstead S-73* (WTU);	AF244716	AY266236	AY562948
***Solanum adscendens***Sendtn.	Brazil	*Stehmann 6001* (BHCB)	JN542580	JN661818	JN661835
***Solanum arboreum*** Dunal	Costa Rica	*Bohs 2521* (UT)	AF244719	DQ180424	AY996381
***Solanum argentinum***Bitter & Lillo	Argentina	*Bohs 2539* (UT)	AF244718	DQ180425	AY996382
***Solanum armentalis***J.L. Gentry & D’Arcy	Costa Rica	*Bohs 2593* (UT);	JN542581	JN661819	JN661836
***Solanum betaceum*** Cav.	Bolivia	*Bohs 2468* (UT)	AF244713	DQ180426	AY996387
***Solanum celsum***Standl. & C.V. Morton	Costa Rica	*Bohs 2592* (UT)	JN542582	JN661820	JN661837
***Solanum conglobatum***Dunal	Bolivia	*Bohs 2740* (UT)	JN542583	JN661821	JN661838
***Solanum cordovense*** Sessé & Moc.	Costa Rica	*Bohs 2693* (UT)	AF244717	DQ180480	AY996401
***Solanum deflexum***Greenm.	Costa Rica	*Bohs 2715* (UT)	JN542584	DQ180427	DQ169025
***Solanum deflexum***Greenm.	Mexico	*Smith & Rojas 348* (NY)	JN542585	JN661822	JN661839
***Solanum evolvuloides***Giacomin & Stehmann	Brazil	*Jardim 1843* (NY)	JN542586	JN661823	JN661840
***Solanum evolvuloides***Giacomin & Stehmann	Brazil	*Giacomin 974* (BHCB)	JN542587	JN661824	JN661841
***Solanum granuloso-leprosum***Dunal	Paraguay	*Bohs 3190* (UT)	JN542588	JN661825	JN661842
***Solanum hirtellum***(Spreng.) Hassl.	Paraguay	*Bohs 3166* (UT)	JN542589	JN661826	JN661843
***Solanum lepidotum*** Dunal	Costa Rica	*Bohs 2621* (UT)	JN542590	DQ180486	DQ169035
***Solanum lignescens***Fernald	Mexico	*Linares 3536* (MEXU)	JN542591	JN661827	JN661844
***Solanum luteoalbum***Pers.	BIRM S.0042	*Bohs* 2337 (UT)	AF244715	DQ180433	AY562957
***Solanum manabiense***S. Stern	Ecuador	*Stern & Tepe 377* (UT)	JN542592	JN661828	JN661845
***Solanum manabiense***S. Stern	Ecuador	*Stern & Tepe 374* (UT)	JN542593	JN661829	JN661846
***Solanum parcistrigosum***Bitter	Bolivia	*Bohs 2738* (UT)	JN542595	DQ180431	DQ169013
***Solanum parcistrigosum***Bitter	Argentina	*Nee & Bohs 50808* (NY)	JN542597	JN661832	JN661849
***Solanum parcistrigosum***Bitter	Paraguay	*Bohs 3194* (UT)	JN542594	JN661830	JN661847
***Solanum parcistrigosum***Bitter	Brazil	*Aparecida et al. 4860* (NY)	JN542596	JN661831	JN661848
***Solanum pseudocapsicum***L.	BIRM S.0870	no voucher	AF244720	DQ180436	AY562963
***Solanum rugosum*** Dunal	Costa Rica	*Bohs 3011* (UT)	JN542598	DQ180490	DQ169046
***Solanum schlechtendalianum*** Walp.	Costa Rica	*Bohs 2915* (UT)	JN542599	DQ180491	DQ169047
***Solanum turneroides***Chodat	Bolivia	*Nee 51716* (NY);	JN542600	DQ180439	DQ169051
***Solanum umbellatum*** Mill.	Costa Rica	*Bohs 2560* (UT)	JN542601	JN661833	JN661850
***Solanum velutissimum*** Rusby	Bolivia	*Nee 51780* (NY)	JN542602	JN661834	JN661851

## Appendix 2

Aligned dataset. (doi: 10.3897/phytokeys.8.2199.app) File format: NEXUS matrix file.

**Explanation note:** Aligned dataset; alignment performed visually using Se-Al (Rambaut 1996).

**Copyright notice:** This dataset is made available under the Open Database License (http://opendatacommons.org/licenses/odbl/1.0/). The Open Database License (ODbL) is a license agreement intended to allow users to freely share, modify, and use this Dataset while maintaining this same freedom for others, provided that the original source and author(s) are credited.
